# Revisiting Shellfish as the Leading Allergen in Adult-Onset Food Allergy

**DOI:** 10.7759/cureus.94357

**Published:** 2025-10-11

**Authors:** Adam A Kline, Mykhailo Vysochyn, Nicholas Dushenko, Kaden Stewart

**Affiliations:** 1 Medicine, Saint James School of Medicine, The Valley, AIA; 2 Cardiology, Saint James School of Medicine, The Valley, AIA

**Keywords:** adult-onset, adult-onset food allergy, allergy and immunology, allergy to food, fish allergy, ige hypersensitivity, ige-mediated, ige-mediated allergies, shellfish, shellfish allergy

## Abstract

Adult-onset food allergy (AOFA), referring to food allergies that first develop in adulthood, has received growing attention in recent years, reflecting the broader rise of immunoglobulin E (IgE)-mediated food allergies. Despite limited dedicated research, a small but expanding body of observational and clinical data offers insight into its characteristics, particularly the frequency of implicated allergens. In this narrative review, the widely cited claim that shellfish are the most common allergen associated with AOFA is critically examined. To assess this claim, relevant peer-reviewed observational studies, published in English and primarily indexed in the PubMed database between 2000 and 2025, were reviewed. Studies were selected based on their focus on adult populations and the availability of specific allergen data.

Shellfish were consistently identified as one of the most frequently reported allergens in multiple studies conducted across diverse geographic regions. However, substantial variation in study design, population characteristics, diagnostic criteria, and reporting practices hindered direct comparisons. Variability in reported prevalence rates and methodological differences further complicated interpretation. Additionally, limitations such as reliance on self-reported diagnoses and inconsistent use of diagnostic tests were identified as factors that limit the generalizability of current findings.

In summary, although shellfish are frequently reported as a leading allergen in AOFA, current evidence remains inconclusive. Misidentification of the most prevalent allergens could potentially influence clinical guidance and public health interventions. The need for rigorous, large-scale epidemiological studies employing standardized definitions and diagnostic protocols is therefore emphasized. A clearer understanding of allergen prevalence in AOFA will enable more effective clinical risk assessments and support the development of targeted public health strategies.

## Introduction and background

Immunoglobulin E (IgE)-mediated food allergy (FA), while historically seen as predominantly a pediatric condition, is now recognized as a significant and growing concern among adult populations worldwide [1]. In a comprehensive 2023 global cross-sectional study involving 47,572 children and 44,835 adults, Gupta et al. estimated that FA affects between 1% and 10% of the global population [2]. Interestingly, this study also revealed that certain countries, including the United States and Italy, reported a higher prevalence of FA among adults compared to children, challenging longstanding assumptions about the age distribution of this condition. A follow-up analysis of adult participants further classified cases into adult-onset food allergy (AOFA) and childhood-onset food allergy (COFA) based on the reported age of diagnosis [3]. The analysis indicated that AOFA accounted for approximately one-third of all adult FA diagnoses, suggesting that its contribution to the overall burden of FA has likely been underestimated. These findings underscore the growing clinical relevance of AOFA and the need for greater research attention to this underexplored phenomenon.

Despite relevant epidemiological findings such as these, research specifically focused on AOFA remains limited. Few population-based studies have comprehensively examined its unique risk factors, clinical presentations, or long-term outcomes [1]. This lack of dedicated research poses challenges for developing evidence-based guidelines tailored to the prevention and management of AOFA. Currently, the mainstay of FA management in adults, regardless of the age of onset, is strict food avoidance [4]. This approach involves accurately identifying the allergens responsible for triggering IgE-mediated responses and counseling patients to avoid exposure to these triggers [5,6]. Given this management strategy, understanding which allergens most commonly contribute to adult-onset cases holds significant therapeutic relevance for clinicians seeking to improve patient outcomes.

Although limited in scope, the existing literature consistently identifies shellfish as the most frequently implicated allergen in AOFA, surpassing other common allergens such as peanuts, soy, milk, and tree nuts. This observation has important implications for clinical practice, particularly in the areas of risk assessment, diagnostic evaluation, and patient education. However, the validity of this claim requires careful examination, as many of the studies rely on self-reported data or are limited by small sample sizes and potential regional biases. This narrative review aims to critically assess the available evidence regarding the prevalence of shellfish allergy in AOFA, with the goal of informing clinical decision-making and guiding future research toward the development of targeted prevention and management strategies.

## Review

Methodology

**Figure 1:**
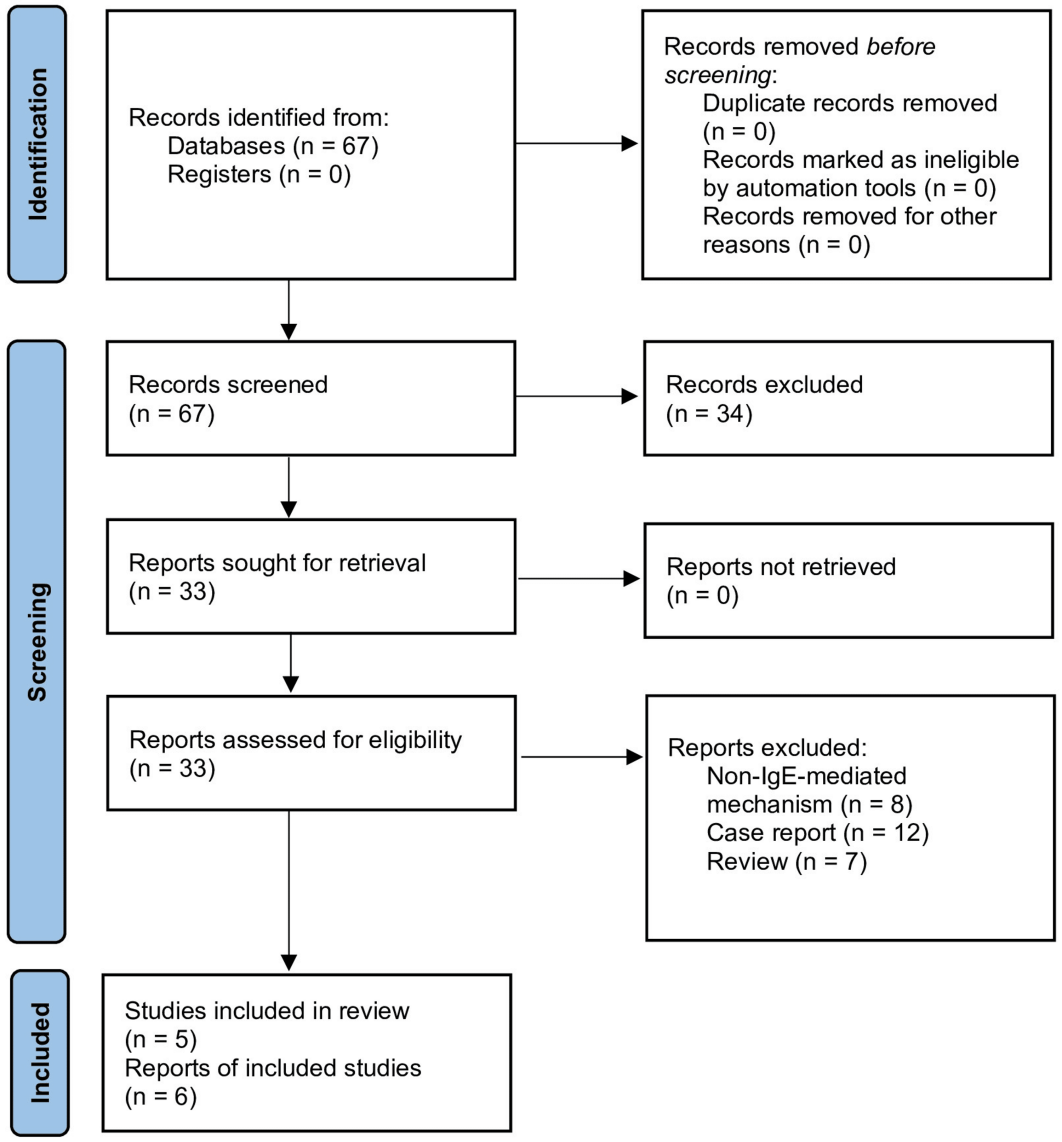
PRISMA (2020) flow diagram of the selection process. PRISMA: Preferred Reporting Items for Systematic Reviews and Meta-Analyses; IgE: immunoglobulin E.

An informal literature search was conducted using the PubMed/National Center for Biotechnology Information (NCBI) database, restricted to English-language publications from the year 2000 onward, to ensure the most recent literature was obtained. As this is a narrative review, and the number of studies being examined is relatively small, an additional database was not deemed to be necessary. The search employed the term “adult-onset AND food AND allerg*”, allowing for the inclusion of variations such as "allergy" and "allergic". Studies were excluded if they met any of the following criteria: (1) narrative review articles or case reports; (2) focus on non-IgE-mediated mechanisms; (3) lack of specific allergen identification; or (4) no apparent relevance to the topic. This search yielded 67 results, of which only six met the inclusion criteria. Figure 1 outlines the selection process, and Table 1 highlights the reports that were ultimately included.

**Table 1:** Final studies selected from PubMed/National Center for Biotechnology Information (NCBI).

Title	Authorship	Year of publication
Prevalence and characteristics of adult patients with adult-onset and childhood-onset food allergy	Sompornrattanaphan et al. [7]	2023
Phenotypic characterization of childhood- and adult-onset food allergy among adults in the United States	Hultquist et al. [8]	2022
Quality of life is lower in adults labeled with childhood-onset food allergy than in those with adult-onset food allergy	Patel et al. [9]	2021
Prevalence and characteristics of peanut allergy in US adults	Warren et al. [10]	2021
Adult-onset IgE-mediated food allergy at a Winnipeg allergy clinic: a case series	Walter and Kalicinsky [11]	2020
Prevalence and characteristics of adult-onset food allergy	Kamdar et al. [12]	2015

It should be noted that this search was exploratory and not exhaustive; therefore, the included studies may not fully represent the breadth of data available on this topic.

Discussion

Nearly all of the studies identified through the PubMed/NCBI search reported findings indicating that shellfish are the most commonly implicated allergen in cases of AOFA. This section will briefly examine each study’s contribution to this observation, highlighting how shellfish were identified as the predominant allergen and noting any relevant contextual considerations. Table 2 briefly summarizes contrasting relevant findings.

**Table 2:** Summary of key findings. AOFA: adult-onset food allergy.

Study	AOFA sample size	Shellfish allergen prevalence (in AOFA population)	Statistical significance	Key limitations
Sompornrattanaphan et al. (2023) [7]	119	59%	No (p = 0.459)	Single center; limited generalizability
Hultquist et al. (2022) [8]	Not explicitly stated	Not explicitly stated	Not explicitly reported	Somewhat inconsistent findings
Patel et al. (2021) [9]	122	28%	No (p = 0.62)	Lacks statistical significance; single institution
Warren et al. (2021) [10]	Not explicitly stated	Not explicitly stated	Yes (p < 0.001)	Not broadly applicable; focused on a specific subgroup
Walter and Kalicinsky (2020) [11]	14	35.7%	Not explicitly reported	Very small sample size; short two-year scope
Kamdar et al. (2015) [12]	171	54%	Not explicitly reported	Relatively small subgroup analysis

Sompornrattanaphan et al. (2023)

The 2023 study by Sompornrattanaphan et al. [7] analyzed data collected between 2009 and 2019 from the Division of Allergy and Clinical Immunology at Siriraj Hospital. Among 119 adults diagnosed with AOFA, 70 individuals (approximately 59%) reported shellfish as the trigger for their initial allergic reaction. However, a univariate logistic regression analysis produced a p-value of 0.459 and an odds ratio of 0.75 (95% CI: 0.35-1.58), indicating that this association was not statistically significant.

These findings suggest that, despite the high observed prevalence of shellfish-triggered reactions within this cohort, the association lacks statistical robustness and may not be generalizable to broader populations. Furthermore, the study’s conclusions are limited by its relatively narrow geographic and demographic focus, which further constrains the applicability of its results.

*Hultquist et al.​​​​​​​ (*​​​​​​​*2022)*

The 2022 study by Hultquist et al. [8] analyzed data from a cross-sectional, population-wide survey of 40,443 US adults conducted between 2015 and 2016. Shellfish were identified as the most prevalent allergen associated with AOFA, affecting approximately 1.99% of the US adult population when adjusted for scale, with prevalence increasing with age. However, it is noteworthy that among adults aged 18-29 years, milk was more commonly implicated than shellfish, and overall, milk allergy prevalence was only slightly lower at 1.77%.

In comparing AOFA and COFA, shellfish showed a proportionally lower association in AOFA relative to COFA when compared to other common allergens. For instance, among adults aged 30-39 years, AOFA attributed to shellfish affected approximately 2% of the population, whereas COFA related to shellfish affected nearly 4.25%, a ratio of 2.125. In contrast, wheat allergy prevalence in the same age group showed a much smaller difference between AOFA (1.35%) and COFA (1.65%), yielding a ratio of about 1.2. While these findings do not negate shellfish’s overall prominence as a leading AOFA trigger, they underscore important distinctions between allergen patterns in AOFA versus COFA.

The statistical significance of these findings was not explicitly reported. Despite this fact and the considerations listed above, the large sample size and broad demographic representation strongly support the association between shellfish and AOFA.

Patel et al.​​​​​​​ (​​​​​​​2021)

The 2021 study by Patel et al. [9] examined survey data from adults who received care at Northwestern Memorial HealthCare clinics between 2002 and 2017. After applying exclusion criteria, 202 adults were included in the final analysis, with 80 classified as having COFA and 122 identified as having AOFA. Among those with AOFA, 28% reported allergic reactions to shellfish, making it the most frequently reported allergen in this group, followed by tree nuts at approximately 20%.

However, the association between shellfish and AOFA was not statistically significant, with a reported p-value of 0.62. In contrast, the association for tree nuts was statistically significant, with a p-value of <0.001. These results suggest that, despite the higher reported prevalence of shellfish reactions, the finding lacks statistical robustness and may be subject to similar limitations as observed in the Sompornrattanaphan study [7], including limited generalizability and potential sample size constraints.

Warren et al.​​​​​​​ (​​​​​​​2021)

Similar to the 2022 study by Hultquist et al. [8], the 2021 study by Warren et al. [10] analyzed data from the 2015-2016 nationwide survey of the US adult population. This study specifically focused on individuals affected by peanut allergy. It found that the likelihood of developing shellfish-associated AOFA varied significantly depending on whether individuals had childhood-onset or adult-onset peanut allergy. Those with adult-onset peanut allergy were nearly five times more likely to have shellfish-associated AOFA compared to those with childhood-onset peanut allergy. Interestingly, the opposite pattern was observed for shellfish-associated COFA, where prevalence was higher among those with childhood-onset peanut allergy. These associations were reported as statistically significant, with a p-value of <0.001.

However, it is important to note that across nearly all subgroups analyzed, tree nuts emerged as the most prevalent comorbidity associated with AOFA, a finding that contrasts with the focus on shellfish. Additionally, because this study concentrated on a specific subgroup, i.e., individuals with peanut allergy, its findings may not be broadly generalizable to the adult population as a whole and offer limited insight into the overall role of shellfish in AOFA.

Walter and Kalicinsky​​​​​​​ (2020)

The 2020 study by Walter and Kalicinsky [11] conducted a retrospective review of patient records from the Winnipeg Allergy and Asthma Clinic between May 2018 and July 2020. Fourteen adults were identified as having adult-onset IgE-mediated food allergy (AOFA), all of whom had previously tolerated the implicated foods. Shellfish were the most commonly reported allergen, implicated in five of the 14 cases (35.7%), followed by finfish in three cases (21.4%), and wheat/flour and pollen-food syndrome in two cases each (14.3%).

While the study underscores the prominence of shellfish as a frequent trigger for AOFA within this cohort, its small sample size and relatively short two-year observational period limit the strength and generalizability of these findings. Consequently, this study provides only limited support for the broader hypothesis that shellfish are the predominant trigger of AOFA across diverse populations.

Kamdar et al.​​​​​​​ (2015)

The 2015 study by Kamdar et al. [12] utilized data from the 2011-2012 National Health and Nutrition Examination Survey (NHANES) to evaluate the prevalence and characteristics of AOFA among a nationally representative sample of 2,719 US adults. Following the application of specific diagnostic criteria, 171 individuals were identified as having AOFA, and this subgroup formed the basis for further analysis.

Within this population, shellfish emerged as the most frequently implicated allergen, associated with 54% of all AOFA cases, followed by tree nuts at 43%. While the study benefits from a large, representative initial sample, the relatively small number of individuals identified with AOFA (n = 171) limits the statistical power of the analysis and the precision of prevalence estimates. This small subpopulation also restricts the ability to perform robust subgroup analyses across demographic variables such as age, sex, and ethnicity. As a result, although the study offers valuable insights into the epidemiology of AOFA, its findings should be interpreted with caution regarding their generalizability to the broader adult population.

## Conclusions

The current body of literature on AOFA consistently identifies shellfish as the most frequently implicated allergen across a limited but diverse group of study populations and research methodologies. However, despite the apparent consistency, critical limitations, including small AOFA-specific sample sizes and limited statistical power, necessitate cautious interpretation of these findings. Although a degree of consensus appears to be emerging regarding the predominance of shellfish in AOFA, the overall generalizability of the evidence remains uncertain due to considerable methodological and diagnostic criteria variability and a lack of standardized outcome measures across studies. These associations remain underexplored and poorly understood, limiting the ability to provide targeted prevention or treatment strategies.

Future investigations should prioritize large-scale, longitudinal studies that employ rigorous, standardized diagnostic protocols and objective testing methods to more accurately define the epidemiology, risk factors, and clinical outcomes associated with AOFA. These studies should also ensure the inclusion of diverse populations and settings to enhance the generalizability of findings. Moreover, integrating clinical, immunological, and genomic data will be essential to uncover the biological mechanisms driving adult-onset sensitization and to distinguish AOFA from childhood-onset allergies that persist into adulthood. Improved data synthesis, consensus on case definitions, and attention to social determinants of health will further strengthen the evidence base. These efforts are essential to inform targeted public health interventions, refine clinical management strategies, and ultimately improve outcomes for the growing population of adults affected by this increasingly recognized and clinically significant condition.
